# Concentration-related metabolic rate and behavioral thermoregulatory adaptations to serial administrations of nitrous oxide in rats

**DOI:** 10.1371/journal.pone.0194794

**Published:** 2018-04-19

**Authors:** Karl J. Kaiyala, Douglas S. Ramsay

**Affiliations:** Department of Oral Health Sciences at the University of Washington, Seattle, WA, United States of America; Oregon Health and Science University, UNITED STATES

## Abstract

**Background:**

Initial administration of ≥60% nitrous oxide (N_2_O) to rats evokes hypothermia, but after repeated administrations the gas instead evokes hyperthermia. This sign reversal is driven mainly by increased heat production. To determine whether rats will behaviorally oppose or assist the development of hyperthermia, we previously performed thermal gradient testing. Inhalation of N_2_O at ≥60% causes rats to select cooler ambient temperatures both during initial administrations and during subsequent administrations in which the hyperthermic state exists. Thus, an available behavioral response opposes (but does not completely prevent) the acquired hyperthermia that develops over repeated high-concentration N_2_O administrations. However, recreational and clinical uses of N_2_O span a wide range of concentrations. Therefore, we sought to determine the thermoregulatory adaptations to chronic N_2_O administration over a wide range of concentrations.

**Methods:**

This study had two phases. In the first phase we adapted rats to twelve 3-h N_2_O administrations at either 0%, 15%, 30%, 45%, 60% or 75% N_2_O (n = 12 per group); outcomes were core temperature (via telemetry) and heat production (via respirometry). In the second phase, we used a thermal gradient (range 8°C—38°C) to assess each adapted group’s thermal preference, core temperature and locomotion on a single occasion during N_2_O inhalation at the assigned concentration.

**Results:**

In phase 1, repeated N_2_O administrations led to dose related hyperthermic and hypermetabolic states during inhalation of ≥45% N_2_O compared to controls (≥ 30% N_2_O compared to baseline). In phase 2, rats in these groups selected cooler ambient temperatures during N_2_O inhalation but still developed some hyperthermia. However, a concentration-related increase of locomotion was evident in the gradient, and theoretical calculations and regression analyses both suggest that locomotion contributed to the residual hyperthermia.

**Conclusions:**

Acquired N_2_O hyperthermia in rats is remarkably robust, and occurs even despite the availability of ambient temperatures that might fully counter the hyperthermia. Increased locomotion in the gradient may contribute to hyperthermia. Our data are consistent with an allostatic dis-coordination of autonomic and behavioral thermoregulatory mechanisms during drug administration. Our results have implications for research on N_2_O abuse as well as research on the role of allostasis in drug addiction.

## Introduction

To identify the thermoregulatory and motivational consequences of nitrous oxide (N_2_O) administration in rats, we have conducted a wide range of studies involving the use of direct and indirect calorimetry, temperature telemetry, thermal gradients, place preference, N_2_O self administration and stress hormone release [1–15]. To date, our work has primarily involved clamped administrations of ≥60% N_2_O.

When first administered at these levels N_2_O increases heat loss and causes hypothermia [3, 5, 6, 8, 9, 11, 13, 16]. Following several administrations, core temperature instead remains within the normal ‘homeostatic’ range during administration–the hallmark of hypothermic tolerance–but this tolerance mostly results from an acquired increase of metabolic rate. More remarkably, with further administrations the rats exhibit hyperthermia, hypermetabolism and elevated heat loss during N_2_O administration [6, 8, 9, 11, 13]. We regard this situation as an instance of allostatic—not homeostatic—regulation involving an energetically inefficient overcorrection of a regulated outcome [17]. Moreover, the switch from initial hypothermia to eventual hyperthermia represents a clear and multiply replicated instance of a ‘sign-reversal’ [18], a phenomenon of particular interest in pain and addiction research because the chronic use of pain-reducing drugs such as morphine can make those drugs acquire pain-promoting properties (e.g., opioid-induced hyperalgesia [19]) and because the chronic use of recreational drugs with initially pleasurable effects can make those drugs induce negative affective and hedonic properties that perversely become major drivers of addiction [20, 21]; reviewed in [17].

When given the choice of thermal conditions in a thermally graded alleyway during 60% N_2_O inhalation, rats selected cooler ambient temperatures both during initial administrations when hypothermia occurred, and also during subsequent administrations when hyperthermia occurred [7, 11]. The combination of hypothermia and cool-seeking behavior during initial administration might be interpreted as a ‘regulated hypothermia’ [22] that serves a protective function because hypothermia reduces the harmfulness and lethality of toxicants [23]. However, the combination of acquired intra-administration hyperthermia with a persisting preference for cooler ambient temperatures [11, 12] represents an acquired dis-coordination between autonomic and behavioral effectors, another proposed feature of allostatic regulation [12, 17] that may represent a basis for the transition to a variety of disordered biobehavioral disease states such as drug addiction and obesity [10, 12, 17].

We previously reported that initial administrations of 30% or 50% N_2_O did not alter core temperature, but did increase heat loss; core temperature homeostasis was preserved owing to simultaneous increases in heat production [5]. These data demonstrate that drug administrations that have no effect on core temperature may nonetheless have significant biological effects on the physiological and behavioral determinants of core temperature–a lesson that emphasizes the interpretational challenges associated with using core temperature in toxicology screens [24]. Similarly, some rats are insensitive to 60% N_2_O’s typical hypothermic effect during initial administration because increased heat loss is offset by a prompt increase of heat production [3, 13].

Importantly, rats that exhibited minimal hypothermia during initial 60% N_2_O administration engaged in significantly more N_2_O self-administration in comparison to hypothermia-sensitive rats, and the minimal hypothermia animals also exhibited elevated metabolic heat production in a final test session in the calorimeter [13]. These data indicate that a robust (but usually hidden) regulatory phenotype can explain what looks like initial drug insensitivity as defined by the relative stability of core temperature during drug administration (core temperature has long used to define and study drug sensitivity and tolerance in animal models [4, 10, 25, 26], and has also been used to screen for toxicants [24]). This perspective suggests a “reactive” explanatory basis for the well-documented finding that humans who appear relatively insensitive to alcohol upon initial administration based on easily measured but complexly determined “distal” outcomes are at greater risk for subsequently developing drug use disorders than are their more initially sensitive counterparts [27, 28]. Lower initial sensitivity to the antinociceptive effect of morphine is also a predictor of opiate addiction in rats [29]. The point is that “initial insensitivity” to a drug of abuse may not be what it appears to be (true insensitivity), but can instead mask a regulatory hyper-reactiveness that participates in the addictive process.

‘Dose-response’ data are critically important for understanding the drug’s broader spectrum of physiological and behavioral actions, and are of major value for designing studies aimed at specific questions of interest. Therefore, in the present study, groups of rats with telemetric temperature implants were adapted in a calorimeter to 12 administrations of N_2_O at one of six concentrations ranging from 0% to 75% and then subsequently tested at their assigned N_2_O concentration in an instrumented thermally graded alleyway to measure behavioral temperature preference, locomotion and core temperature.

## Materials and methods

### Subjects and ethics approval

Male Long-Evans rats (Charles River, N = 72, ~21 d of age upon arrival) were maintained in an AAALAC-accredited facility on a 12h:12h light/dark cycle (lights on at 0700 h) at an ambient temperature of 22 ± 1°C. Rats were group-housed in polycarbonate tubs with unrestricted access to water and pelleted chow (5053 PicoLab Rodent Diet 20, Animal Specialties and Provisions, Quakertown, PA). All animal procedures were approved by the University of Washington Institutional Animal Care and Use Committee.

### Surgery

A telemetric temperature sensor was implanted surgically into each rat’s peritoneal cavity under isoflurane anesthesia (3–5% for induction and 1–3% for maintenance) while the rat was on a 39°C heating pad. Meloxicam (an NSAID) was provided in the drinking water (0.02 mg/ml H_2_O) from 1 day before to 2 days after surgery. Rats were ~ 28–30 d of age at surgery. Rats were allowed to recover for at least 7 d after surgery before beginning studies.

### Design and procedures

In brief, this study involved two phases: phase 1 consisted of 12 3-h N_2_O administrations at one of six concentrations during which heat production and core temperature were measured in calorimeters maintained at the accustomed housing temperature (22 ± 1°C); phase 2 consisted of testing the same rats one time in a thermally graded alleyway during which core temperature, selected ambient temperature and locomotion distance were measured during a 3-h exposure to the same N_2_O concentration as in phase 1. The gradient allows the animals to choose ambient temperatures ranging between 8 and 38°C. These exposures commenced at the same time as the calorimetry exposures (see below). Phase 1 was intended to quantify thermoregulatory adaptations to serial N_2_O exposures [6, 8] involving N_2_O concentrations for which no prior serial adaptation studies exist, while phase 2 was designed to jointly assess thermal preference behavior, locomotion and core temperature when the animals were in the drug adapted state. For phase 1, rats (n = 12 per group by random assignment) received 12 N_2_O administrations in a calorimeter at a concentration of 0%, 15%, 30%, 45%, 60%, or 75%. The duration of each calorimeter session was 5-h, consisting of a 2-h control gas baseline (0100-1200h) followed by 3-h N_2_O delivery that commenced at noon (1200-1500h). Sessions occurred on M, W and F. Core temperature (measured via telemetry) and heat production (estimated via respirometry) were obtained during the calorimetry sessions throughout the baseline period and for the 3-h of N_2_O exposure. For phase 2 of the study, each rat received its assigned N_2_O concentration during a 3-h interval that commenced at noon in the course of being housed in a thermally graded alleyway for 24h; rats were placed in the gradient at 4pm (1600h) on the day prior to the day upon which the 3-h N_2_O session occurred.

### Calorimetry and N_2_O systems

Our calorimetry system and gas delivery systems are described in detail elsewhere [3, 6, 8]. In brief, six gas-tight calorimetry systems equipped for telemetric core temperature assessment served as the N_2_O exposure chambers (6.86 liter volume). The N_2_O gas concentrations were composed of 21% oxygen (O_2_) and the assigned % of N_2_O with the balance being nitrogen (N_2_). Gas mixtures were made from medical grade N_2_O, O_2_ and N_2_ using a mass flow controller for each gas with the blend delivered to each chamber at a constant flow rate of 1.5 liters/min. Heat production was quantified based on oxygen consumption using an equation originally derived by Weir [30] that represents an attractive way to accurately estimate heat production when carbon dioxide (CO_2_) production is not measured (N_2_O interferes with CO_2_ measurement). The equation is:

Heat production (W) = 349 × fractional O_2_ delta × incurrent gas flow rate (liters/min) where the fractional O_2_ delta is the difference in the fractional O_2_ concentrations between the incurrent and excurrent gas streams and the gas flow rates are adjusted to standard pressure and temperature, dry. The basis for the validity of this equation is explained in [30–34]. In brief, it works because the error in O_2_ consumption (VO_2_) that would accompany the use of VO_2_ (liters/min) = fractional O_2_ delta × incurrent gas flow rate (liters/min) when the respiratory quotient (RQ, the ratio of CO_2_ production to O_2_ consumption) does not equal one is almost exactly cancelled by the energy liberated per liter of O_2_ consumed at the actual RQ value in effect.

The first calorimeter exposure session began ≥ 7 d after surgery and recovery. Mean body mass at first exposure was 155.9 ± 23.42 (SD) g.

### Thermal gradient

This study employed two identical custom-built gas-tight thermal gradients (described in detail [7, 11, 12] based on Gordon’s design [35, 36]. In brief, a water jacket on each end of the gradient enables two recirculating water baths to pump warm water to one end and cool water to the other, creating a temperature continuum ranging from 8°C to 38°C along the length of the gradient’s insulated copper shell. A removable acrylic alleyway (182.9 cm long, 12.06 cm high, 12.06 cm wide) is suspended from an internal support structure within the thermal gradient. The rat’s location in the alleyway is detected using 24 infrared beams that are placed 19 mm above the alleyway floor and positioned 76.2 mm apart along the length of the alleyway ending 38.1 mm from either end. A thermistor with 0.1°C accuracy is positioned 10 mm above each of the 24 infrared transmitters and measures the ambient temperature at the rat’s location. An antenna made from two continuous 22 gauge solid strand insulated wires surrounds the lateral walls of the alleyway and receives the telemetric core temperature signal from a sensor within the rat’s peritoneal cavity. Uniform illumination during the light portion of the 24-h light/dark cycle is provided by 128 evenly spaced white light LEDs that run the length of the alleyway and project their light upward from top edge of the internal support structure. Pelleted chow and water were freely available in the center of the alleyway. Room air was purified, dehumidified and compressed to provide the control gas, and was delivered to the thermal gradient at a flow rate of 10 liters/min. The N_2_O gas concentrations were made as described above and delivered to the thermal gradient at a flow rate of 10 liters/min. Concentrations of N_2_O, O_2_, and CO_2_ were measured using an infrared gas analyzer that sampled gas via a T-connector placed in the incurrent and excurrent gas lines connected to the gradient’s copper shell.

### Telemetry, data acquisition and instrument control

Telemetric measurement of core temperature was accomplished using a commercial system from Data Sciences International (Saint Paul, MN) that consists of a Data-Exchange Matrix, Physio-Tel Receiver (Model RPC-1), Dataquest ART 4.2 software, and an implantable battery-powered temperature sensor (model TA-F40) implanted in the rat’s peritoneal cavity. The antenna system used in each direct calorimeter consists of two radio ferrite coils oriented perpendicularly to each other and epoxied underneath a Plexiglas platform that holds them 2 mm above the floor of the direct calorimeter. The antennae wires exited the calorimeter through a gas-tight port and were connected to the RPC-1 receiver base. The antenna wires located in the thermal gradient are exteriorized through a sealed port in the copper shell and are connected to the RPC-1 receiver base by adding second wire wrappings to the receiver base’s two ferrite coils so that the external wire antenna can conduct its radio signal to the receiver. All other instrument control and data acquisition were performed using custom programs written in LabVIEW 6.8 (National Instruments, Austin, Texas).

### Data reduction

For phase 1 (the 12 calorimetry sessions), the dependent variables were core temperature and heat production. Core temperature was recorded at 15 s intervals while heat production data were recorded at 10 s intervals. For depictions of within-session time plots, mean core temperature and heat production were calculated for each 6-min bin. For statistical analyses, calorimeter outcomes were analyzed as changes (Δ) from baseline values that were defined as the means of the final 12 min of the baseline period. Statistical analyses were performed on averaged change data (means) across each half of each 3-h administration period.

For phase 2 (thermal gradient testing), ambient temperature at the rat’s selected position within the gradient was logged at the time the rat’s position was recorded. The rat’s position was recorded at 7-sec intervals based on 24 infrared beams that were spaced 7.62cm apart along the length of the alleyway. Distance travelled was computed as the average value of the difference between successive time-stamped rat-position values multiplied by 7.62 cm. Distance was summed within each 6-min bin. Selected ambient temperature was calculated as the mean temperature of the thermistor(s) corresponding to the interrupted infrared beam location(s). Core temperature data were recorded at 30-s intervals. For within-session time plots, mean core temperature and mean selected ambient temperature values were computed within each 6-min time bin. For statistical analyses of gradient data, core temperature and selected ambient temperature for each rat were each averaged (means) across each half of the 3-h administration period. As the animals were placed in the gradient the day before measurements, baseline was defined as the mean value over the 60 min prior to N_2_O onset. To compute total distance in the thermal gradient studies, individual binned distance data were summed across each half of the N_2_O exposure. Distance data and estimates of energy costs of locomotion were summarized as median, 25^th^ percentile and 75^th^ percentile values due to skewness and unequal variances between dose groups.

### Locomotion energy cost estimates

To estimate individual energy costs of locomotion (E in units of W/kg), distance within each 6-min bin was converted to mean speed (S) in units of m/s and entered into the formula for the minimal energy cost of transport (mCOT) [37]:

$$\frac{E}{M}=10.7M^{-0.316}S$$

(We use E instead of HP for thermal gradient locomotion metabolic rate estimates because a substantial fraction of the total energy cost of locomotion (>25% [38]) is not expressed as heat, whereas trivial locomotion occurs in calorimeter studies due to limited space). Thus HP was estimated as 75% of E. In estimating locomotion costs we excluded the term corresponding to the energy cost due to the sum of the cost of support (COS) and resting metabolic rate (RMR) (given as the intercept value in [37]) because our system does not allow resolving whether the rat is standing or not. This is important because COS + RMR represents an important contributor to the gross energy cost of locomotion (= mCOT + COS + RMR). We predicted the effect of mCOT on Tcore by summing estimates of mCOT and assuming that rats had a specific heat of 3.47 J/(g°C) [39].

### Statistical analyses

The first two 6-min bins of heat production data following time zero were omitted from analysis due to the potential for artifactual values in this interval [3]. To jointly assess the effects of N_2_O concentration, session and the concentration by session interaction, the correlated longitudinal calorimetry data were analyzed using the linear mixed-model program in SPSS Statistics 24 (IBM, Somers, NY). Session and dose were treated as fixed effects, and change scores were adjusted for baseline. First-order autoregressive covariance structures were specified for longitudinal analyses [40]. For comparisons between concentration groups within each session, the GLM univariate approach in SPSS was employed; 95% confidence intervals and p-values were adjusted for baseline values. Core and selected ambient temperature in the gradient were analyzed with adjustment for baseline using the GLM univariate procedure in SPSS. Distance data were analyzed for significance using Mood’s median test in SPSS Statistics v. 24.

For within-session time plots of absolute heat production in the initial vs. final exposure N_2_O sessions, heat production was adjusted for body mass, an important determinant of metabolic rate [41], which increased over the course of the 12 exposure sessions. Because heat production during N_2_O inhalation is affected by a complex and incompletely understood array of drug effects and biobehavioral control system adaptations, the adjustment for heat production was based on the coefficient estimate identified by the regression of baseline pre-exposure heat production on body mass across the 12 N_2_O exposure sessions using linear mixed model analysis to account for the correlated nature of the within-subject data. Specifically, adjusted heat production (HP) was calculated as follows:

HPadjusted,ij=HPij+b(M¯−Mij)b=coefficientidendifiedfromregression=0.0022HPij=individualrat'sunadjustedHPinsessionjHPadjusted,ij=individualrat'sadjustedHPinsessionjM¯=grandmeanofbodymassacrossall12exposuresessions=259.7gMij=individualrat'sbodymassinsessionj

## Results

### Phase 1–repeated N_2_O administrations

**Fig 1:**
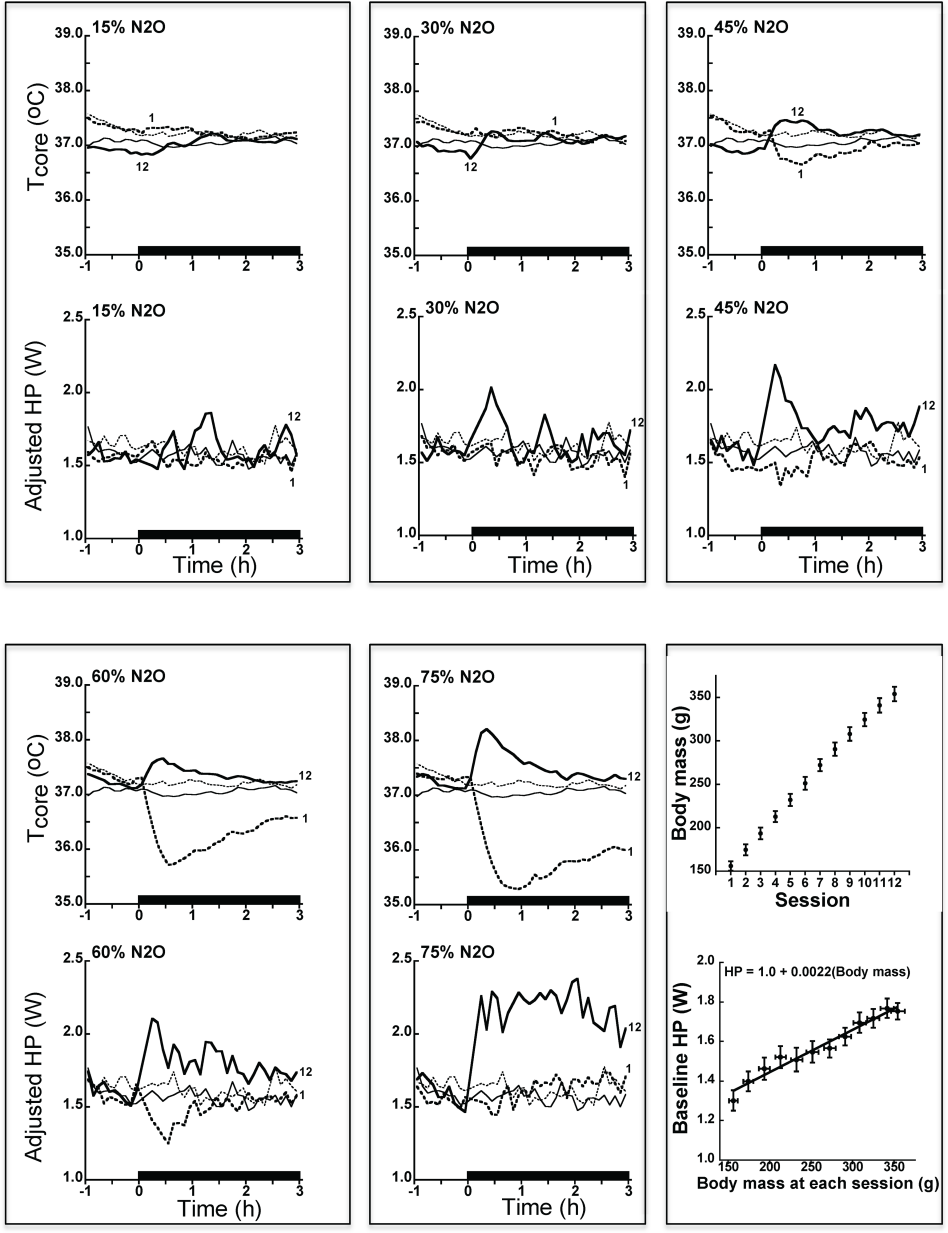
Temporal profiles depicting concentration-dependent thermal adaptations to 12 steady-state nitrous oxide exposures in rats. The time plots depict mean core temperature and body-mass adjusted heat production in the first and twelfth 3-h N_2_O exposure sessions (indicated by the numbers in each concentration group). Bars indicate period of N_2_O inhalation. Thin lines depict outcomes during control gas (custom control air, o% N_2_O) administration; dashed thin line denotes first control session, solid thin line, denotes twelfth control session). N = 12 per dose group. During the course of the calorimetry studies, body mass increased by ~125% whereas baseline HP increased by ~35% (error bars are 95% confidence intervals). The regression equation for HP was used to adjust HP for body mass in the time series plots (see Materials and Methods). N_2_O, nitrous oxide; Tcore, core temperature; HP, heat production.

**Fig 2:**
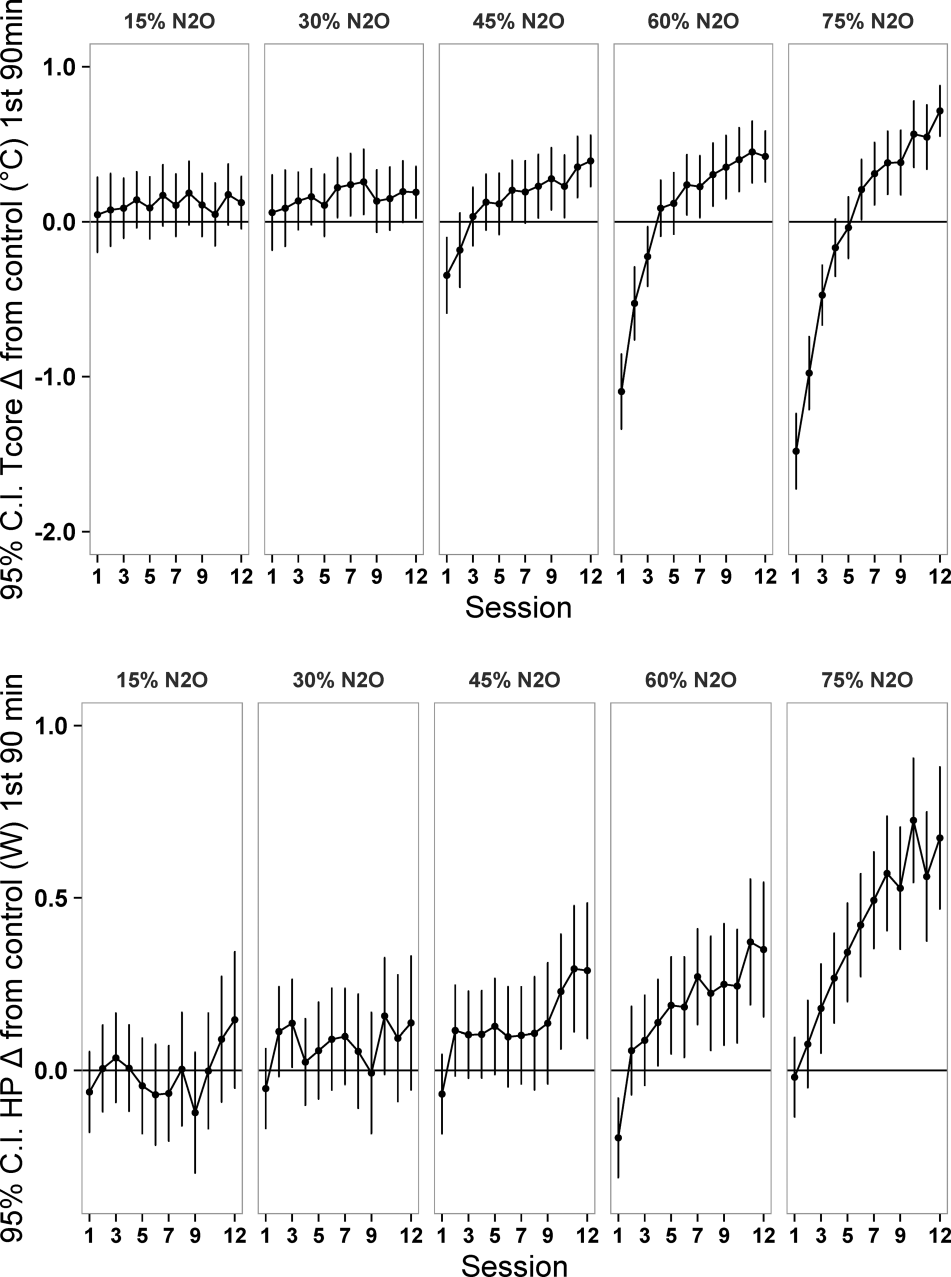
**Nitrous oxide minus control gas differences in core temperature and heat production in each of the twelve 3-h exposure sessions (first 90 min).** 95% confidence intervals that do not include zero are significantly different from control at p<0.05. N = 12 per dose group. C.I., confidence interval; N_2_O, nitrous oxide; Tcore, core temperature; HP, heat production.

**Fig 3:**
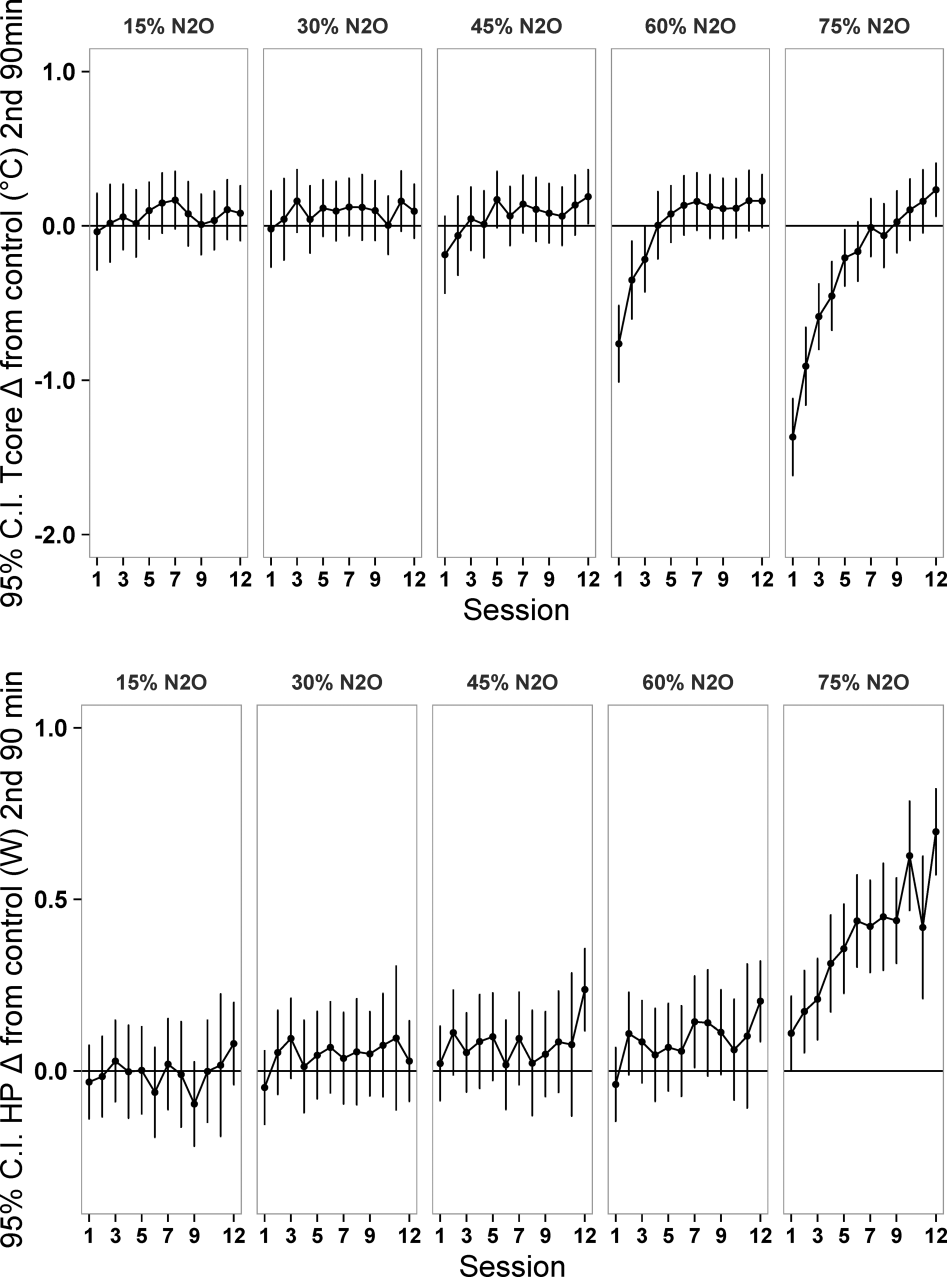
Nitrous oxide minus control gas differences in core temperature and heat production in each of the twelve 3-h exposure sessions (second 90 min). For explanation see legend for Fig 2.

As predicted, phase 1 resulted in substantial thermal adaptations over the course of the 12 administrations of N_2_O (Figs 1, 2 and 3; Figs 2 and 3 depict mean changes of the N_2_O-treated rats from the control rats while S1 and S2 Figs depicts mean changes from baseline.). Mixed model analysis of core temperature data averaged over the first 90 min of administration indicated highly significant effects of session and the concentration by session interaction (p<0.0001), but the main effect of concentration was non-significant (p = 0.20), consistent with the core temperature sign-reversals from hypothermia to hyperthermia in the three highest concentration groups (particularly evident in Fig 2). For heat production, the effects of drug concentration and session were highly significant (p<0.0001), and there was a significant concentration by session interaction (p = 0.004). The magnitudes of the thermal adaptations from the first to the twelfth administrations depicted in Fig 2 are worth noting for the three highest concentration groups: core temperature values increased (mean ± SE) by 0.75±0.111°C (45% N_2_O), 1.47±0.116°C (60% N_2_O), and 2.18±0.199°C (75% N_2_O); all p<0.0001. Corresponding heat production values increased by 0.24±0.062 W (45% N_2_O), 0.50±0.089 W (60% N_2_O), and 0.63±0.146 W (75% N_2_O); p≤0.003.

In the second 90 min of drug administration, the pattern of results for core temperature is an attenuated version of that in the first 90 min in that departures from normothermia were generally of lesser magnitude (compare Figs 2 and 3). Nonetheless, mixed model analysis of core temperature data averaged over the second 90 min indicated highly significant effects of session and the concentration by session interaction (p<0.0001) as well as a significant main effect of concentration (p = 0.0002). For heat production, all terms were highly significant (p<0.0001).

### Phase 2—Thermal gradient test

**Fig 4:**
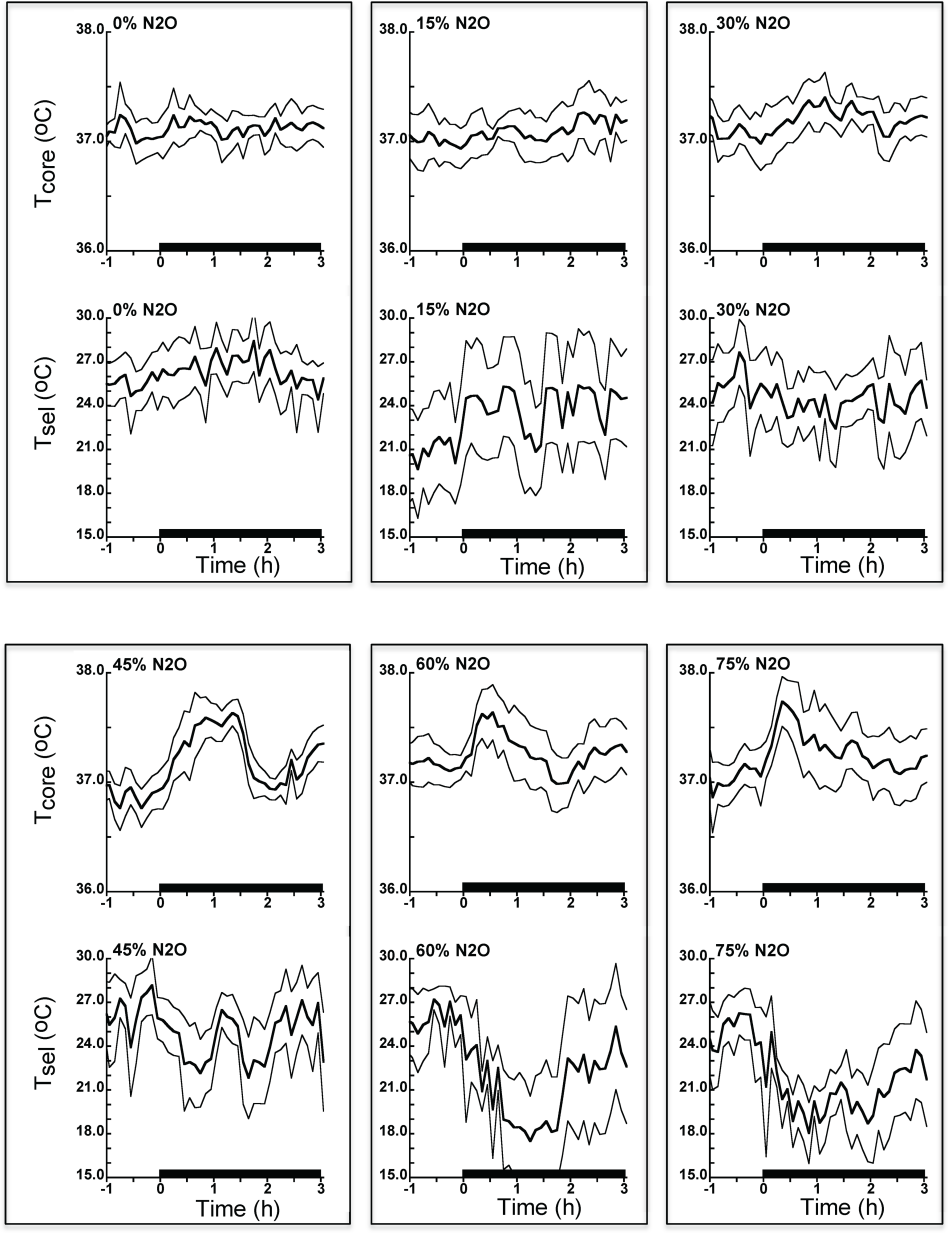
Time series plots of core temperature and selected ambient temperature during a single nitrous oxide exposure in the thermally graded alleyway following 12 drug administrations in the calorimetry phase of the study. Despite access to ambient temperatures that presumably would have prevented hyperthermia (8°C at the coldest end), rats in the higher dose groups selected only modestly lower temperatures and still developed hyperthermia in the earlier part of administration. N = 12 per group. Data are mean ± 95% confidence limits. Tcore, core temperature; Tsel, selected ambient temperature; N_2_O, nitrous oxide.

**Fig 5:**
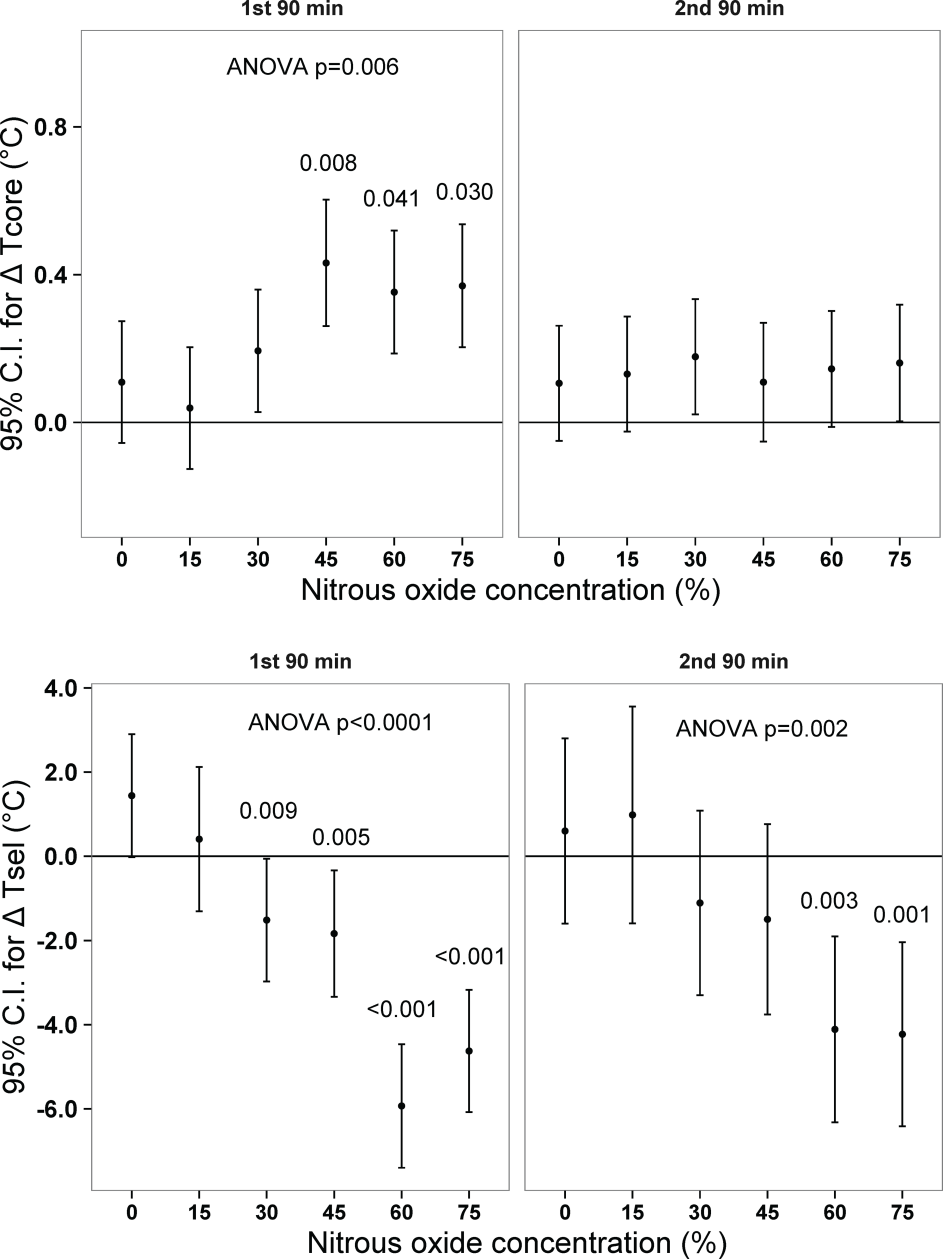
Analysis of baseline-adjusted mean changes in core temperature and selected ambient temperature during nitrous oxide exposure in the thermally graded alleyway. 95% confidence intervals that do not include zero are significantly different from baseline at p<0.05. Numbers above error flags are p-values for the group differences from the control gas condition. N = 12 per group. Overall baseline core temperature was 37.0°C (95% C.I. = 36.9, 37.1) while baseline selected temperature was 24.9°C (95% C.I. = 24.3, 25.7). Tcore, core temperature; Tsel, selected ambient temperature; N_2_O, nitrous oxide; C.I. confidence interval.

To assess the impact of behavioral thermoregulation on core temperature during N_2_O administration in the adapted state, the rats were tested once in the thermally graded alleyway at their assigned % N_2_O. As depicted in Figs 4 and 5, N_2_O administration at concentrations of ≥45% elicited significant hyperthermia relative to the control gas condition while ≥30% N_2_O evoked reliable hyperthermic changes relative to baseline. Note that the hyperthermic changes occurred despite reliable decreases in thermal preference for ≥30% N_2_O, indicating incomplete behavioral compensation for the effect of presumed increases of heat production that mediates hyperthermia.

In the second 90 min, none of the concentration groups exhibited mean changes in core temperature that were reliably different from control (Fig 5). However, both the 60% and 75% groups exhibited reliable decreases in selected temperature, suggesting that this behavioral outcome helped normalize core temperature.

**Fig 6:**
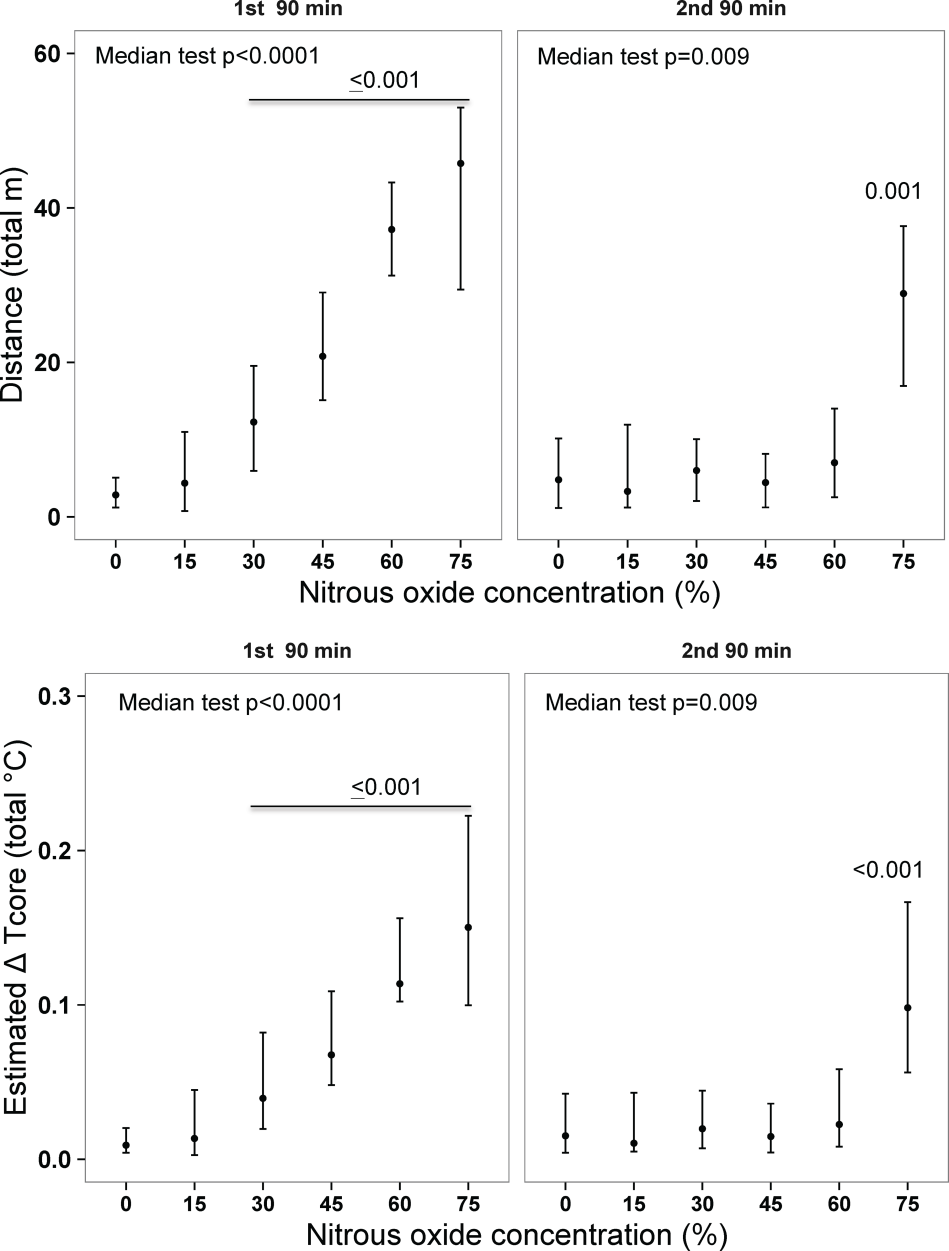
Concentration-related stimulatory effect of nitrous oxide on total locomotion and its estimated influence on core temperature during thermal gradient testing. Data are medians bounded by 25^th^ and 75^th^ percentiles. P-values for comparisons with control gas condition are indicated. Estimated changes in core temperature are based only on minimum cost of transport and ignore the influence of resting metabolic rate and the cost of support (see Materials and Methods); hence the total contribution of locomotion to body heat content is probably higher. N = 12 per group. Tcore, core temperature.

Importantly, our data revealed a highly significant dose-related stimulatory effect of N_2_O on total locomotion in first 90-min of N_2_O administration (Fig 6).

This finding raises the possibility that the heat byproduct of locomotion contributes to an increase of core temperature (and so offsets the effect of reduced selected temperature to promote heat loss). Consistent with this possibility are the estimated total effects on core temperature made by the minimum cost of transport (mCOT; see Materials and Methods) (Fig 6). Note that this analysis underestimates the gross thermal contribution of locomotion, which also includes a non-trivial but unknown contribution from resting metabolic rate plus an increment caused by the cost of support (see Materials and Methods). In a further exploration of this issue, linear mixed model analysis of the time series data revealed that locomotion distance was indeed a highly significant positive predictor of core temperature in a model that included both N_2_O concentration and distance (1^st^ 90 min: estimated change in Tcore = 0.032±0.0068°C per m of locomotion; p<0.0001; 2^nd^ 90 min: estimated change in core temperature = 0.029±0.0075°C per m; p = 0.0001). Interestingly, after accounting for the independent effect of distance, the omnibus effect of N_2_O concentration *per se* was non-significant (1^st^ 90 min, p = 0.07). Another indication that locomotion may have been a major driver of hyperthermia is that core temperature was essentially normothermic and statistically indistinguishable across all except the highest concentration groups in the 2^nd^ 90 min of administration (Fig 5) when activity had dropped off and become similar among the zero through 60% N_2_O groups (Fig 6). The essential point is that locomotion in the thermal gradient may have contributed to the hyperthermia observed in that setting (whereas limited space in the calorimeter setting greatly limits locomotion).

## Discussion

We performed the current study to gain further insight into the nature of the acquired metabolic rate and behavioral adaptations made in response to serial drug administrations using our thermoregulatory models [3, 7]. Key strengths include steady-state administration of a minimally metabolized gas [42] to simplify data interpretation by eliminating pharmacokinetic and metabolite-based explanations; telemetry to preclude handling and stress confounding; and continuous measurements of a ‘high priority’ homeostatic variable [43] along with its underlying determinants using calorimetry (e.g., heat production) [3] and thermal choice via thermal gradient testing [7]. As thermoeffector loops are proposed to work in a relatively independent fashion [44, 45], an important aim in the present study was to assess not just the regulatory response magnitudes but also the functional directions of the metabolic heat production and behavioral thermoregulatory responses to N_2_O administration following chronic N_2_O inhalation sessions among six concentration groups. Accordingly, we documented core temperature and heat production across twelve 3-h N_2_O administrations at concentrations ranging from 0% to 75% N_2_O; and we then measured each adapted group’s thermal preference and locomotion along with their relationship to core temperature during N_2_O inhalation at the accustomed concentration in a thermally graded alleyway. Our findings extend previous knowledge involving high N_2_O concentrations (≥60%) by demonstrating that serial N_2_O administrations to rats at concentrations as low as 30% promote dose-related hyperthermic intra-administration states indicative of thermal allostasis, and that the animals will behaviorally oppose the acquired hyperthermic states by selecting cooler ambient temperatures when tested in the thermal gradient. Yet the extent to which the cooler ambient temperature behavioral response countered the hyperthermic core temperature effect was incomplete on its face, as core temperature during the first 90-min of N_2_O inhalation was reliably above baseline for ≥ 30% N_2_O and was reliably higher than the control group’s for ≥ 45% N_2_O. Our working interpretation of this result is multifactorial as the present work demonstrates that N_2_O inhalation in adapted rats stimulates locomotion in the thermal gradient in a dose-related fashion. As such, locomotion-based heat production in the thermal gradient (discussed below) might combine with another source(s) of heat production that occurs in the limited confines of our calorimeters to cause hyperthermia. We hypothesize that the ‘other source’ involves stress-related neurochemical signaling. Consistent with this proposition, we recently found that acute 60% N_2_O administration at normal laboratory temperature in Plexiglas chambers stimulated corticosterone, epinephrine, and norepinephrine [15], key neuroendocrine effectors of the hypothalamic-pituitary-adrenal (HPA) and sympathetic nervous system (SNS) -mediated biobehavioral stress responses. One of these responses, moreover, was reported to be a type of non-shivering thermogenesis observed during conditioned fear and mediated by beta-adrenergic signaling, but which does not involve interscapular brown adipose tissue (iBAT) [46]. Congruent with the latter finding, we previously found that serial administrations of 60% N_2_O in Plexiglas chambers led to the hyperthermic sign-reversal but did so in the absence of a role for iBAT as documented by both telemetry and infrared thermography [14], pointing to a thermogenic mechanism of unknown origin. This work and related data highlight two important and underappreciated concepts that are, 1) a number of tissues other than BAT are capable of non-shivering heat production (including white adipocytes under stimulation by the sympathetic nervous system; interspersed among white adipose tissue, these cells are also named 'beige' or 'brite' cells [47–49]); and, 2) the SNS can activate thermogenic tissues in a selective fashion [46, 50]. We note also that while the limited space in our calorimeters and Plexiglas chambers does limit locomotion, increased standing and rearing can nonetheless occur and so might contribute to elevated heat production in those settings. Therefore, a future goal should be to better distinguish between the roles of purely facultative vs. behavioral sources of elevated heat production during drug administrations or other interventions such as conditioned fear testing in relatively confined spaces.

The present study builds upon previous work from our group and highlights issues that do not align well with the standard homeostatic model of regulation. In our first thermal gradient study [7] naïve rats tested once in the thermal gradient during 60% N_2_O inhalation exhibited substantial hypothermia (~2°C) in combination with a marked decrease of selected ambient temperature (~10°C). This result seemed commensurate with the concept of “regulated hypothermia” [22] and it seemed appropriate to interpret those results as such [7]. This initial work naturally begged the question of whether rat would behaviorally assist the hyperthermic sign reversal once that was established (an acquired “regulated hyperthermia”), although we thought it more likely that the rats would use behavior to facilitate tolerance development and oppose the development of hyperthermia. To test that hypothesis [12], rats were given ten 3-h 60% N_2_O administrations in our thermal gradient apparatus held at a fixed temperature of 21°C and they acquired the hyperthermic sign reversal, just as observed when studied in the calorimeter. In the next test we turned the gradient on and the rats prevented an increase in core temperature by selecting cooler temperatures [12]. This finding suggested that when provided the opportunity to behaviorally counter an acquired allostatic hyperthermia in a familiar environment, rats would do so such that core temperature is restored to its normal tight homeostatic range. But it subsequently became clear that the tendency to acquire the hyperthermic sign reversal is exceedingly robust, because in our next study [11], when rats were adapted to twelve 3-h 60% N_2_O administrations given in the active gradient (ambient temperature range of 8–38°C), they too developed a significant hyperthermic sign reversal–and did so while consistently selecting cooler ambient temperatures. Thus, while it seems clear that N_2_O-adapted rats can prevent hyperthermia by using behavior [12], the broader picture now indicates that the long-term biobehavioral adaptation to a wide range of N_2_O concentrations favors the acquisition of intra-administration hyperthermia even when the animals are provided with the opportunity to completely obviate its development by selecting sufficiently cool ambient temperatures. Indeed it is worth emphasizing that, theoretically, the animals could have prevented hyperthermia because the coldest pole of our thermal gradient is 8°C. Accordingly, it is noteworthy that in the first 90 min of N_2_O administration, the 60 and 75% N_2_O groups changed average thermal preference values by a seemingly modest amount, from 25–26°C (approximately thermoneutral for rats [51]) to “only” about 20–21°C, i.e., to values not much different then the fixed 22°C ambient temperature in the calorimeter studies. But exactly what this means in terms of heat transfer is difficult to say, firstly because those animals did intermittently select substantially cooler temperatures (e.g., they went below 12°C 10% of the time) whereas control animals in the 0% N_2_O group almost completely avoided temperatures below 21°C (<5% of the time). Secondly, it is inadvisable to equate thermal conditions between the calorimeter and gradient environments based on ambient temperature alone, because the ‘weightings’ of convection, conduction, radiation and evaporation likely differ in those environments. The point is that the determinants of body heat balance during N_2_O inhalation are complex and conditioned on the environment in which testing occurs. Emphasizing this, the present work identified an unexpected mechanism for hyperthermia in the thermal gradient, because we now recognize that heat production via increased locomotion might be an important driver of hyperthermia in that environment. This hypothesis is supported both by calculations involving the energy cost of transport, and by the highly significant correlation between locomotion and core temperature during gradient testing. Indeed, when N_2_O dose group was adjusted for locomotion, adjusted core temperatures were similar between groups (p = 0.07), suggesting that were it not for the concentration-related locomotion-stimulating effect of N_2_O, the relatively modest degree of cool-seeking behavior may have been sufficient to more completely prevent hyperthermia in the higher drug concentration groups. At this point our working hypothesis is that the animals are regulating core temperature at a ‘balance point’ that reflects the net result of increased non-shivering heat production, increased locomotion and augmented heat loss by selecting cooler ambient temperatures. Consistent with the idea that the observed hyperthermia during N_2_O inhalation in the gradient mainly reflected elevated locomotion, a reexamination of the data in our previous active gradient study [11] indicates that the development of hyperthermia proceeded in tandem with the development of increased locomotion (Fig 2 in [11]). In any event, our findings seem difficult to explain based on the concept of homeostasis, and are more compatible with an allostatic view of regulation [10, 17] in which the mismatched concurrent activation of opposing effector responses can result in clear departures from homeostasis.

We have given thought to potential mechanisms of thermal choice behavior during N_2_O inhalation. In one study involving restrained Sprague-Dawley rats, 40% N_2_O inhalation in a warm environment (30°C ambient temperature) increased tail blood flow [52]. If this response occurs in unrestrained Long-Evans rats tested in our thermal gradient, it might tend to motivate a preference for a cooler ambient temperature by warming thermoreceptors in the tail. Arguing against this possibility, however, are the results of a study in our laboratory in which unrestrained and well-habituated Long-Evans rats were evaluated for tail temperature using infrared thermography at 21–22°C ambient temperature [14]. Surprisingly, tail temperature did not increase during any of the nine administrations of 60% N_2_O; in fact, tail temperature actually decreased during the initial administration [14]. This result suggests that N_2_O inhalation does not increase tail blood flow in our study animals and, therefore, suggests that increased tail blood flow is not involved in the N_2_O’s effect on thermal preference.

N_2_O abuse is an underappreciated and growing problem [53]; indeed N_2_O ranks as the second most popular recreational drug in the UK [54]. Whether our findings are relevant to human use of this drug is an important but largely unanswered question. Work involving single cool water immersion sessions during inhalation of 25–30% N_2_O indicated that the drug augmented hypothermia development by lowering the core temperature threshold for activation of shivering thermogenesis [55–57]. However, to our knowledge, the effects of *repeated* N_2_O administrations on thermoregulatory outcomes in humans simply have not been the subjects of published research. A related literature involves alcohol, a drug that shares a number of properties with N_2_O [58–60], including in humans an initial hypothermic effect that can be followed by a hyperthermic sign reversal during prolonged administration [61].

The question of N_2_O’s thermal effects in humans would seem to be of importance because the maintenance of body and brain temperature within narrow limits is of high priority to survival in homeothermic species [43]. Brain temperature is an especially vital homeostatic variable, and brain hyperthermia markedly alters neuronal function including the sensitivity of neurons to neurotransmitters, and increases the toxicological effects of drugs (reviewed in [62]). Body hyperthermia, as observed in the present study, would be expected to increase brain temperature unless the peripheral effect is offset by a reduction of neuronal heat production due to reduced activity, but human data suggests that acute N_2_O inhalation does not alter global cerebral metabolic rate [63]. Accordingly, it seems that the intra-administration body hyperthermia observed in the present study would increase brain temperature. Importantly, however, to our knowledge the effects of repeated N_2_O administrations on brain temperature or intra-brain heat production are completely unknown. If repeated N_2_O administrations cause an increase of brain metabolic rate, then this and the peripheral hyperthermia would presumably be additive and might drive brain temperature to levels with significant neurotoxic implications. This area of inquiry may be of interest given that recreational N_2_O use is reported to be rapidly increasing in a number of settings [54] and often involves chronic use with toxic effects [64]. Indeed, our data indicating that chronic administration of lower concentrations of N_2_O can engender significant systemic temperature effects in rats should foster questions regarding the widespread misbelief that recreational N_2_O abuse is relatively benign. The mistaken notion that N_2_O is a “safe” drug derives from the drug’s long history of use and excellent safety record when administered clinically as a component of anesthesia or as an anxiolytic and analgesic agent in sub-anesthetic concentrations.

N_2_O has both neuroprotective effects (owing to its effect as an NMDA antagonist [65]) and neurotoxic actions, but some work in rats indicates that the neuroprotective effects are outweighed by a homocysteine-mediated neurotoxic cascade that persists long after N_2_O inhalation [66]. One report indicates that prolonged anesthesia with N_2_O acts to cause inflammation of the cerebral microvasculature and brain [67]. Important to note is that these studies evaluated the toxic / impairing effects of N_2_O using procedures in which the drug was co-administered with other anesthetic agents. The important point is that our data indicating that chronic administrations of relatively low concentrations of N_2_O tend to promote hyperthermia in combination with reports implicating N_2_O as a CNS inflammatory agent would seem to add further caution against the notion that N_2_O is a ‘safe’ recreational agent.

The data from this study will be valuable in designing future studies on the behavioral pharmacology of N_2_O. Our previous work primarily has involved N_2_O concentrations of 60% and greater, but the present data indicate robust effects at 45% N_2_O on core temperature and heat production (but a less robust effect on thermal preference) and that even repeated administrations of 30% N_2_O have small but significant thermal effects. The robustness of N_2_O’s thermal effects is underscored by our previous finding that attempts to extinguish the allostatic hyperthermia were not successful [9]. Future research on N_2_O allostasis investigating hyperthermic sign reversals that result from the concurrent activation of opposing autonomic and behavioral effectors should consider using N_2_O concentrations in the range of 45–75% N_2_O. However, the magnitude of the effect sizes for measures of core temperature, heat production and selected temperature suggest that studies using 60%-75% N_2_O are optimal to study dysregulation using this model of drug-induced allostasis.

## Supporting information

S1 Fig Reliabilities and effect sizes of mean changes from baseline evoked by nitrous oxide exposure (first 90 min). These metrics are indicated by 95% confidence intervals for baseline-adjusted changes in core temperature and heat production in the first and second 90 min of each of the 12 3-h exposure sessions. The x-axis depicts selected session numbers but data for all sessions are presented. N = 12 per dose group. Note the concentration related pattern of the first 90 min results during initial and final administrations. Note also that 30% N_2_O evokes significant (p<0.05) acquired changes in the first 90 min, providing evidence for system sensitivity to a N_2_O concentration that might be deemed sub-threshold if based on outcomes during initial administration. N_2_O, nitrous oxide; C.I., confidence interval; Tcore, core temperature; HP, heat production.
(TIFF)

S2 Fig Reliabilities and effect sizes of mean changes from baseline evoked by nitrous oxide exposure (second 90 min). See legend for S1 Fig.
(TIFF)

S1 File Supplement PONE-D-17-23852.pdf (supporting information for S1 and S2 Figs and additional data analysis). (DOCX)
