# Supplementary figures and images for: Concentration-related metabolic rate and behavioral thermoregulatory adaptations to serial administrations of nitrous oxide in rats
Source: PLoS One. 2018 Apr 19;13(4):e0194794. doi: 10.1371/journal.pone.0194794 (PMC5909668; doi:10.1371/journal.pone.0194794)

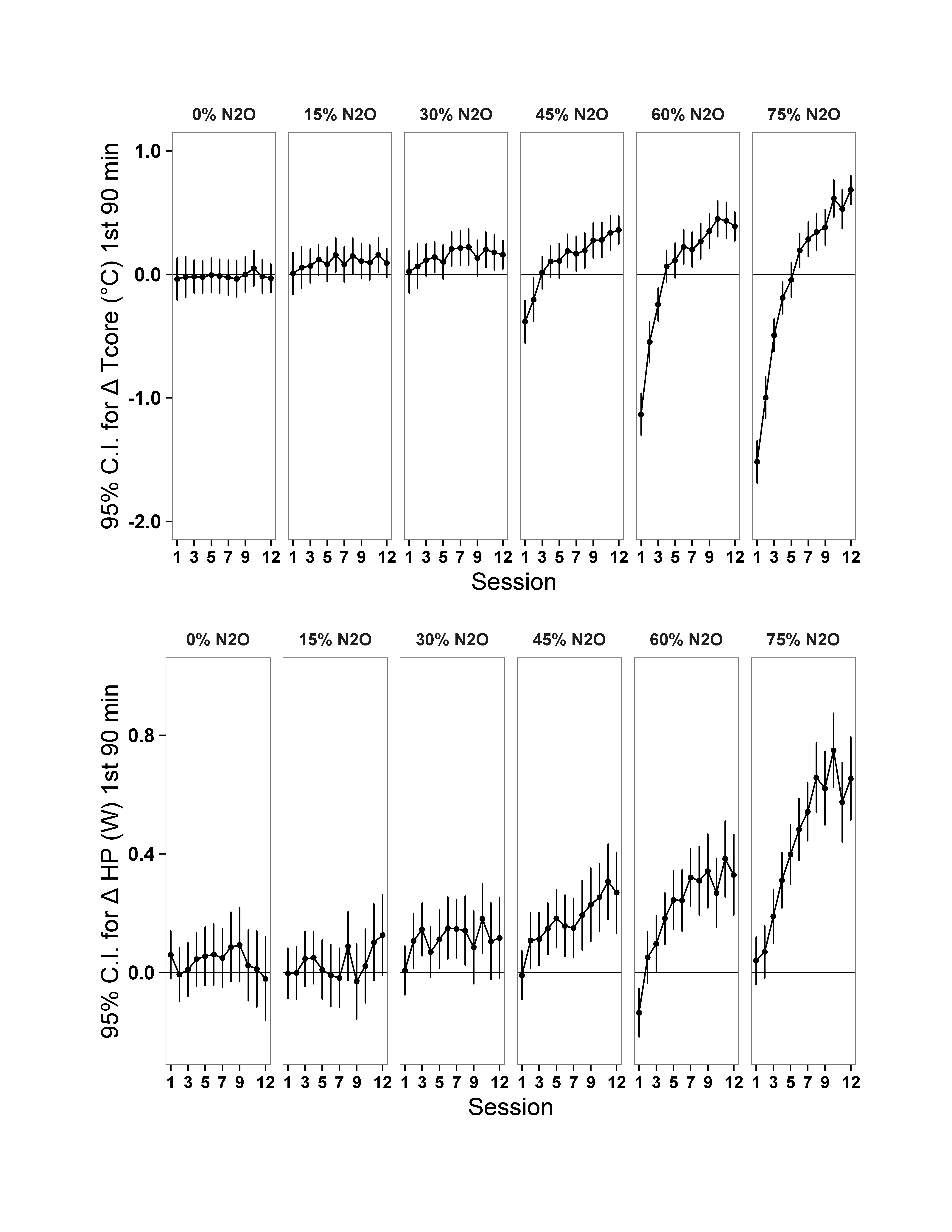

Supplement: S1 Fig — These metrics are indicated by 95% confidence intervals for baseline-adjusted changes in core temperature and heat production in the first and second 90 min of each of the 12 3-h exposure sessions. The x-axis depicts selected session numbers but data for all sessions are presented. N = 12 per dose group. Note the concentration related pattern of the first 90 min results during initial and final administrations. Note also that 30% N2O evokes significant (p<0.05) acquired changes in the first 90 min, providing evidence for system sensitivity to a N2O concentration that might be deemed sub-threshold if based on outcomes during initial administration. N2O, nitrous oxide; C.I., confidence interval; Tcore, core temperature; HP, heat production. (TIFF) [file pone.0194794.s001.tiff]

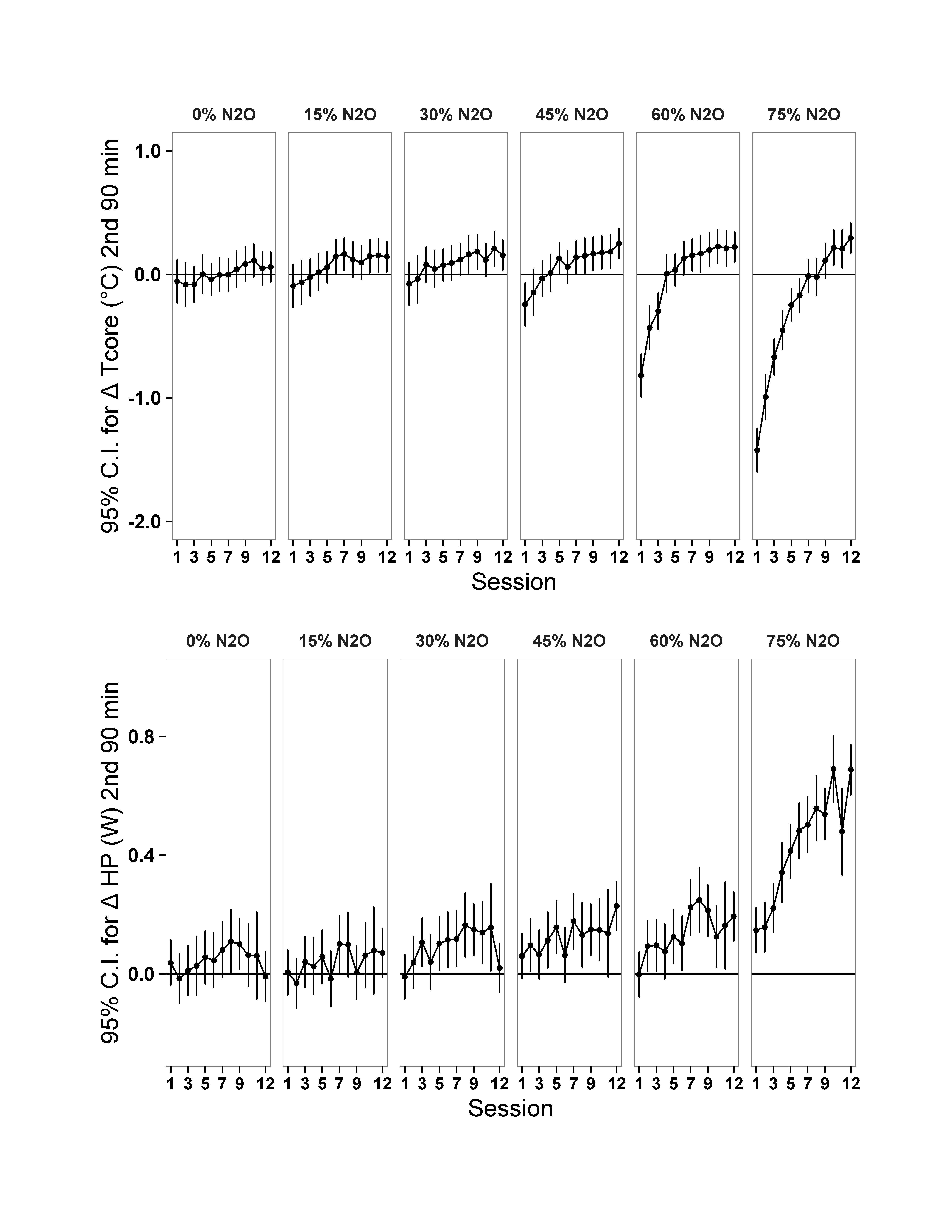

Supplement: S2 Fig — See legend for S1 Fig. (TIFF) [file pone.0194794.s002.tiff]
