# Supplementary material for: Concentration-related metabolic rate and behavioral thermoregulatory adaptations to serial administrations of nitrous oxide in rats
Source: PLoS One. 2018 Apr 19;13(4):e0194794. doi: 10.1371/journal.pone.0194794 (PMC5909668; doi:10.1371/journal.pone.0194794)
Supplement: S1 File — (DOCX) [file pone.0194794.s003.docx]

**Supporting Information for S1 and S2 Figs and Additional Data Analysis**

S1 and S2 Figs depict mean within-session N2O –evoked departures of core temperature and heat production from baseline. Within-session GLM analysis of core temperature in session 1 indicated that linear and quadratic contrasts of the concentration groups were highly significant (p<0.0001). However, in session 1, mean baseline-adjusted delta core temperature differed significantly from the control group only for %N2O ≥ 45% (measures of the effect sizes for the different groups are depicted in Figs 2 and 3 of the main manuscript in terms of 95% confidence intervals). Note that both 60% and 75% N2O evoked a pronounced initial hypothermic effect, and that the hypothermic effect persisted throughout the 3-h exposure (Fig 1 in main manuscript). Interestingly, however, despite the lack of significant hypothermia during initial exposure in the 15% and 30% N2O groups, our data more broadly indicate that the rats were nonetheless sensitive to the drug at these lower doses. Mixed model analysis encompassing all 12 administrations indicated small but significant hyperthermic effects of dose in the first 90 min period for both the 15% group (mean core temperature 0.106±0.029 (SE) °C higher than control; p=0.002) and the 30% group (mean core temperature =0.150±0.049 °C higher than control; p =0.006). The effect of 15% N2O to promote a subtle state of hyperthermia in the first 90 min in the 15% and 30% N2O groups was not accompanied by reliably increased heat production (p=0.91 for 15%; p = 0.09 for 30%), suggesting the recruitment of a subtle heat conserving response in these groups.

The hypothermia evoked upon initial exposure in the three highest concentration groups was attended by an inconsistent heat production response. Heat production was not reduced in the 45% or 75% groups, but was reliably decreased in the 60% group, an effect observed in previous work by our group ([1](#_ENREF_1)). Thus, consistent with previous work, N2O promotes hypothermia primarily via an increase in the heat loss rate relative to the heat production rate, but a reduction in heat production may also contribute to negative heat balance during 60% N2O inhalation.

Core temperature and heat production adapted to repeated N2O administrations in a dose-related manner characterized the development of hypermetabolic intra-administration phenotypes (Figs 1, 2, 3 and 4 in main manuscript). As may be readily appreciated in S1 and S2 Figs and Figs 2 and 3, the pattern of thermal adaptations to N2O embodied significant and substantial concentration by session interactions such that the steepness and magnitude of the hyperthermic and hypermetabolic adaptation trajectories were clearly functions of the N2O concentration for %N2O ≥45%.

**Reference for Supporting Information**

1. Al-Noori S, Ramsay DS, Cimpan A, Maltzer Z, Zou J, Kaiyala KJ. Brown adipose tissue thermogenesis does not explain the intra-administration hyperthermic sign-reversal induced by serial administrations of 60% nitrous oxide to rats. J Therm Biol. 2016 Aug;60:195-203. PubMed PMID: 27503733.
